# Novel technique for repairing posterior medial meniscus root tears using porcine knees and biomechanical study

**DOI:** 10.1371/journal.pone.0192027

**Published:** 2018-02-06

**Authors:** Jia-Lin Wu, Chian-Her Lee, Chan-Tsung Yang, Chia-Ming Chang, Guoan Li, Cheng-Kung Cheng, Chih-Hwa Chen, Hsu-Shan Huang, Yu-Shu Lai

**Affiliations:** 1 Department of Orthopedics and Traumatology, School of Medicine, College of Medicine, Taipei Medical University, Taipei, Taiwan; 2 Department of Orthopedics, Taipei Medical University Hospital, Taipei, Taiwan; 3 Orthopaedic Devices Research Center, National Yang-Ming University, Taipei, Taiwan; 4 Institute of Biomedical Engineering, National Yang-Ming University, Taipei, Taiwan; 5 Bioengineering Laboratory, Department of Orthopedic Surgery, Massachusetts General Hospital and Harvard Medical School, Boston, MA, United States of America; 6 Graduate Institute for Cancer Biology & Drug Discovery, College of Medical Science and Technology, Taipei Medical University, Taipei, Taiwan; Kanazawa University, JAPAN

## Abstract

Transtibial pullout suture (TPS) repair of posterior medial meniscus root (PMMR) tears was shown to achieve good clinical outcomes. The purpose of this study was to compare biomechanically, a novel technique designed to repair PMMR tears using tendon graft (TG) and conventional TPS repair. Twelve porcine tibiae (n = 6 each) TG group: flexor digitorum profundus tendon was passed through an incision in the root area, created 5 mm postero-medially along the edge of the attachment area. TPS group: a modified Mason-Allen suture was created using no. 2 FiberWire. The tendon grafts and sutures were threaded through the bone tunnel and then fixed to the anterolateral cortex of the tibia. The two groups underwent cyclic loading followed by a load-to-failure test. Displacements of the constructs after 100, 500, and 1000 loading cycles, and the maximum load, stiffness, and elongation at failure were recorded. The TG technique had significantly lower elongation and higher stiffness compared with the TPS. The maximum load of the TG group was significantly lower than that of the TPS group. Failure modes for all specimens were caused by the suture or graft cutting through the meniscus. Lesser elongation and higher stiffness of the constructs in TG technique over those in the standard TPS technique might be beneficial for postoperative biological healing between the meniscus and tibial plateau. However, a slower rehabilitation program might be necessary due to its relatively lower maximum failure load.

## Introduction

Meniscal tears are common injuries caused by the combination of tissue degeneration and high mechanical loads [1]. Anterior and posterior meniscal root tears are becoming increasingly recognized as inducing joint degradation, and greater emphasis has been placed on restoring the meniscal integrity [2, 3]. With growing knowledge during recent decades about the physiological functions of knee joint menisci, the connection between the anterior and posterior roots of each meniscus and the tibial plateau is either by root attachments or direct insertion [4–6]. A tear of the posterior medial meniscus root (MMR; PMMR) is defined as a radial tear located within 10 mm of the posterior root attachment site of the medial meniscus, which was shown to decrease the contact area and increase the contact pressure of the affected compartment [7]. In the field of posterior meniscus root tear surgery, the integrity of the meniscus roots is critical for the ability of the meniscus to absorb hoop stress and prevent meniscal extrusion, and its functions include load transmission, shock absorption, proprioception, and joint stability [8–12].

In 1991, a tear of the posterior root of the medial meniscus was first described in a 20-year-old football player [13], and the incidence and prevalence of PMMR tears have recently increased dramatically [14–18]. The previous report by Choi et al showed that the magnetic resonance imaging could provide >90% diagnostic rate [19]. Biomechanical studies analyzing the effects of the MMPRT on the load transmission capacity of the knee reported that the incidence of medial meniscus posterior root tear (MMPRT) is almost 4-times more prevalent than that of the lateral meniscus, and MMPRT may account for around 20%~30% of medial meniscus tears that appear to be PMMR tears [20, 21]. MMPRTs exhibit a similar extent of increasing contact surface and decreasing peak pressure through loss of hoop tension caused by disruption of circumferential fibers, as those that occur in the total meniscectomized knee [16, 18, 22, 23]. On the other hand, it was suggested that the incidence of MMR avulsion ranges 10.1%~27.8% [11, 24]. Recent studies found that possible causes of PMMR tears are deep squats with a floor-based lifestyle in the elderly and traumatic events in the young [25–28]. Since Kim et al. [16] and Seo et al. [29, 30] showed that full restoration of the knee joint function after pullout suture repair of PMMR tears was difficult to achieve, relatively few articles regarding avulsion fracture of the medial meniscus have been published [27, 31]. Furthermore, meniscal tears are the most common injury of the knee joint, and the posterior horn is the most frequent location. LaPrade et al. [32] indicated that nonanatomic repair at all testing angles resulted in a 44% reduction in the contact area and corresponding increases in the mean and peak contact pressures of 67% and 59%, respectively, compared to an intact knee condition. With the use of cadavers and Fuji sensors to evaluate the contact area and peak contact pressure of the knee joint at different flexion angles, Allair et al. [22] showed that PMMR tears significantly increase the contact pressure by about 25% and reduce the contact area similar to those seen with a total meniscetomy.

A 5-year prospective multicenter follow-up report by Krych et al. [33] for conservative treatment of PMMR tears showed that 87% of patients failed, while 31% of patients underwent total knee replacement at about 30 months. Many surgical treatment options exist, including beneficial clinical results of surgical repair for PMMR tears either using suture anchors or a transtibial pullout suture (TPS) technique [7], which has become popular in recent years [29, 34–36]. Suture anchor technique showed significant lower displacement, higher stiffness, and maximum load without significant difference, compared with TPS [7]. Taking all the biomechanical studies into consideration, in 2012, Moon et al. [36] indicated that extrusion of the medial meniscus would still progress over time. Furthermore, Hong et al. showed that biological healing between the undersurface of the medial meniscus and tibial plateau after repair using pullout sutures in rabbit models was not promising [37]. Chung et al. also showed that severe chondral lesions, varus deformities, and an older age were poor prognostic factors for PMMRT repair, especially modified outerbridge classification grade 3 [38].

Although previous research showed that radial tears of the meniscus root area could leave a gap that makes surgical repair difficult [39, 40], partial meniscectomies which often give rise to knee arthritis are the only treatment for this condition [41, 42]. Based on previous anatomic studies [4, 5, 32], the PMMR is attached to the tibial plateau by a ligamentous structure, and we know of no previous research that conducted such a study to repair PMMR tears using porcine knees. Studying the medial compartment is important, because the medial meniscus is more commonly torn than the lateral meniscus [43], and hence we designed a novel technique to reconstruct the PMMR attachment ligament using a flexor tendon graft (TG). The aim of the study was to compare biomechanical properties of the new TG and the standard TPS technique when applied to PMMR reconstruction using porcine knees. We hypothesized that the TG technique would provide superior biomechanical properties compared to the TPS technique.

## Materials and methods

### Specimen preparation

Fresh porcine hind limbs and porcine knee joints from adult hybrid Landrace-Yorkshire-Duroc pigs were obtained from a local slaughterhouse. In total, 12 porcine tibiae with intact medial meniscus roots were randomly assigned to two groups (*n* = 6 each). The mean age of specimens was 28~30 weeks, and the average weight was 130 kg. The knees were harvested and stored at -20°C after dissection and thawed for 24 h at room temperature before testing. Skin and all muscles were removed. The tibia was disconnected from the femur and cut axially. The femur site was removed after cutting the connecting ligaments and soft tissue, and the tibia site was left intact. The tibia was truncated approximately 15 cm below the joint line. Then, a careful examination of all experimental knees was performed to exclude specimens for which the medial meniscus root was of poor quality. Therefore, six fresh flexor digitorum profundus tendons from the limbs were selected for TGs. Each graft was trimmed to a mean size of 150 mm long and 3 mm in diameter. Prior to testing, all specimens in the TG group were sutured with no. 2 Ethibond sutures (Ethicon, Somerville, NJ, USA) with a whipstitch that promoted uniform loading across each limb during testing.

### PMMR repair techniques

**Fig 1:**
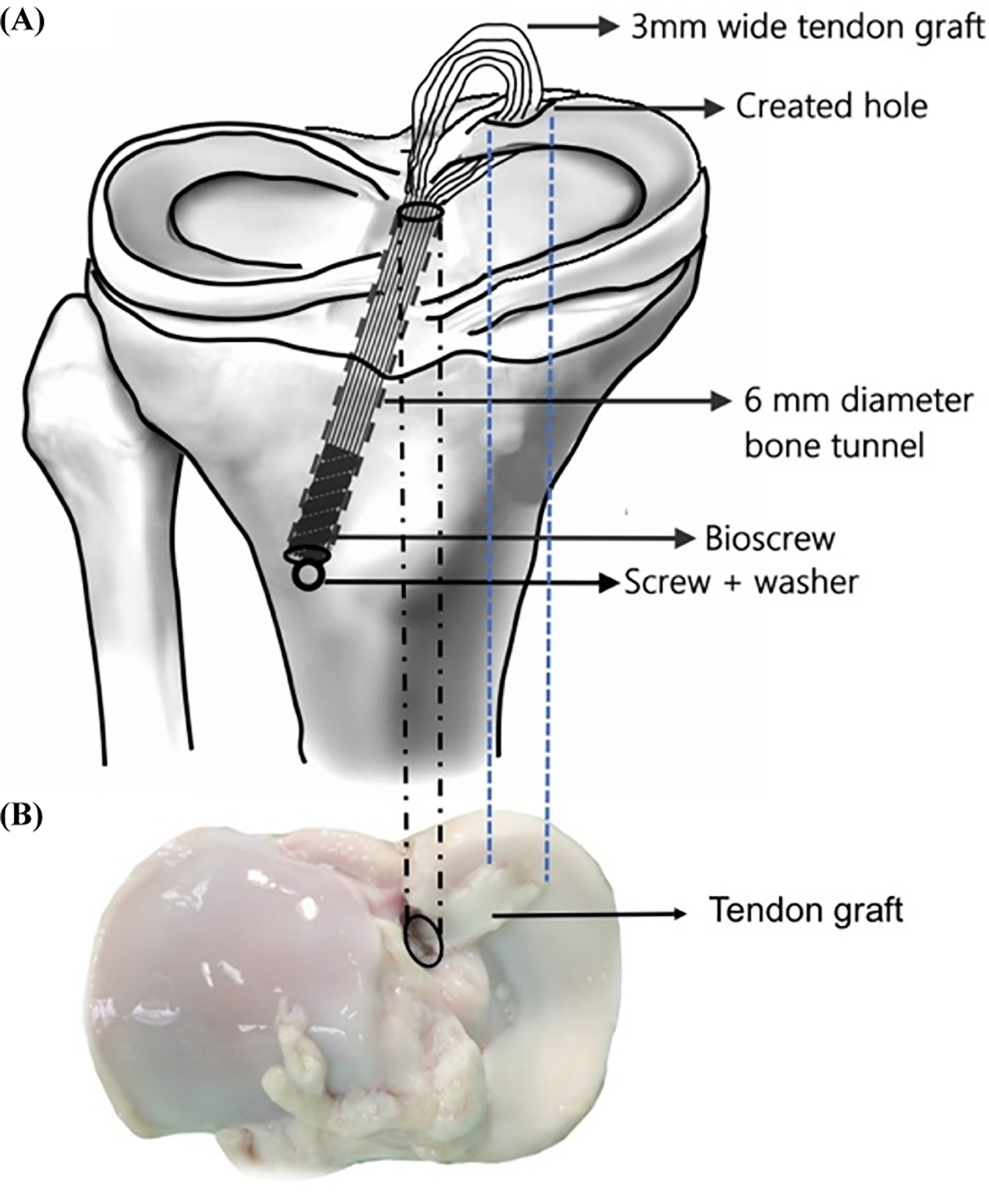
(A) Illustration of the surgical technique for repair of the posterior medial meniscus root (PMMR) using a tendon graft. The posterior horn of the medial meniscus root was reconstructed with a 3-mm-diameter fresh flexor digitorum profundus tendon using a transosseous pullout technique. (B) Photograph demonstrating the complete constructs in the transverse plane.

A single orthopedic surgeon trained in sports medicine carried out all surgical techniques to ensure consistency. A standardized PMMR tear model was created by cutting the peripheral margin of the medial meniscus root area with a 5-mm gap left near the insertion site of the PMMR using a scalpel. The surgical technique of the TG group is shown in Fig 1A. All specimens were tested according to the protocol by Feucht et al. [7] The loading profile consisted of five consecutive phases: (1) a 5-mm longitudinal incision was made through the red-red zone of the posterior medial meniscus with around 5 mm from the root tear site using a no. 15 blade scalpel; (2) a transtibial tunnel was drilled using a 6-mm coring reamer (Arthrex, Karisfeld, Germany) and was aimed at the native medial meniscus root insertion area by an anterior cruciate ligament (ACL) aimer; (3) a prepared fresh flexor digitorum profundus tendon was smoothly passed through the created incision using a suture passer; (4) the graft was threaded through the joint surface, pulled out of the tibial cortex through the 6-mm diameter bone tunnel, and then secured with a 6x25-mm bioscrew (Inion Hexalon^TM^, Tampere, Finland) under 2 N preload; and (5) the graft was augmented with a screw and washer at the anterolateral aspect of the tibia (Fig 1B). For the TPS group, a modified Mason-Allen suture technique was applied to the PMMR tear with no. 2 FiberWire. The sutures were then pulled out through the 5-mm diameter bone tunnel and fixed to the anterolateral aspect of the tibia using a screw and washer [7, 44].

### Biomechanical study

**Fig 2:**
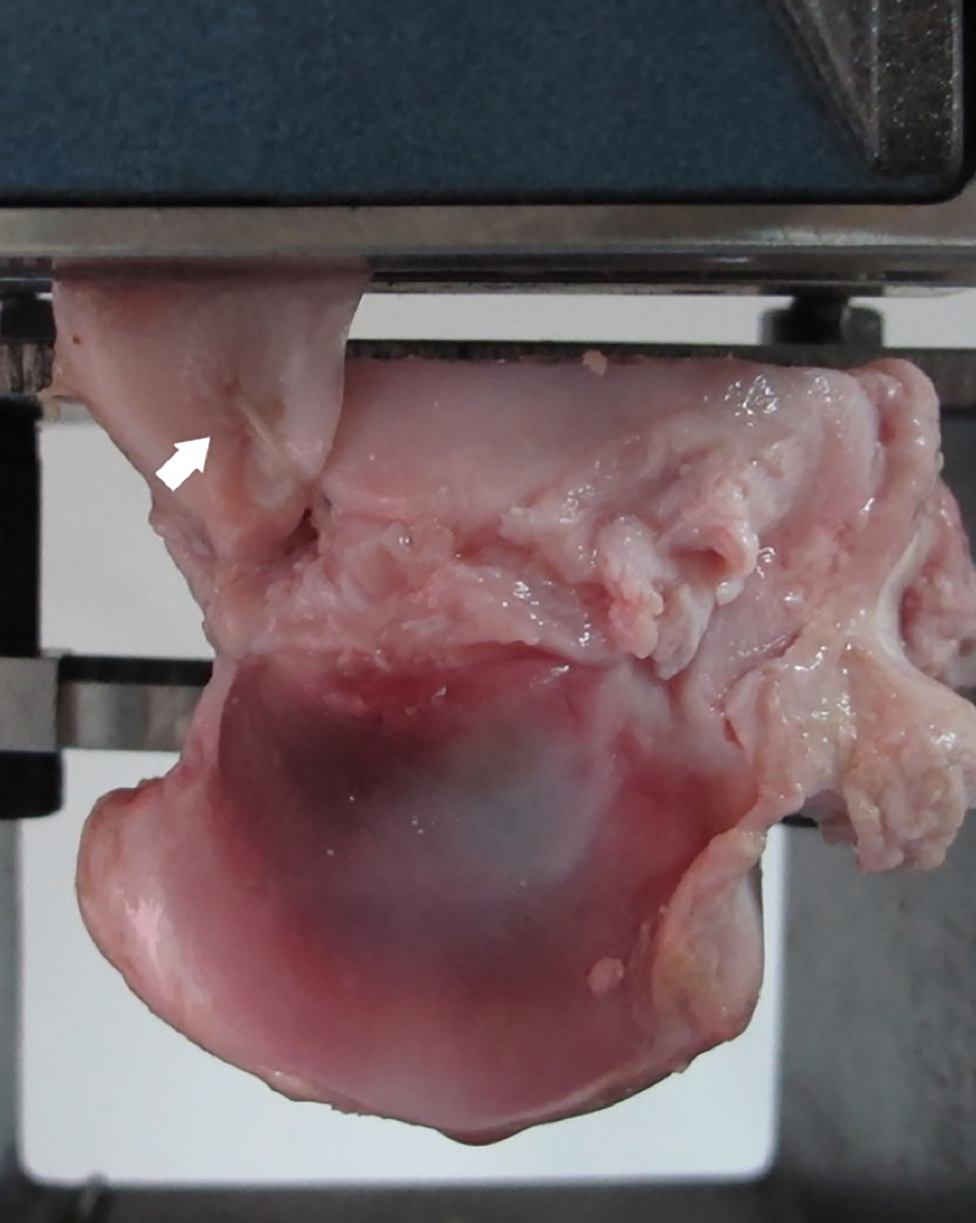
Biomechanical test setup. The clamp was equipped with corrugated jaw faces to prevent meniscus slippage, and the menisci were clamped medial to the sutures or grafts, respectively. The arrow indicates the modified Mason-Allen suture repair.

All tests were performed at room temperature (25 ± 1°C), and specimens were kept moist with saline solution. A material testing system (MTS Bionix 858, Eden Prairie, MN, USA) with a custom-made clamping device was used for tensile testing (Fig 2). The clamping device was rigidly mounted onto the plate of the material testing machine, and the peripheral section of the medial meniscus was placed in a mechanical screw action clamp. In order to prevent meniscus slippage, the clamp was equipped with corrugated jaw faces [7]. To avoid interference with the stiffness analysis, the menisci were clamped 1 cm medial to the sutures or the TGs of the meniscus. After a preload of 2 N, all specimens were subjected to 1000 cycles of a load between 2 and 20 N at a rate of 0.5 Hz. Subsequently, specimens were loaded to failure at a rate of 0.5 mm/s [7]. The number of cycles, displacement, and loads were recorded by MTS software. The following parameters were analyzed in all tests: (1) displacements after 100, 500, and 1000 cycles and (2) the maximum load, stiffness, and elongation at failure load. The displacement was defined as the differences in the crosshead position from the peak of the first cycle to the peak of cycle 100, 500, and 1000. The stiffness was calculated as the steepest slope of the load-deformation curve spanning 30% of the data points collected between load initiation and the maximum load at failure. Elongation was measured as the total displacement of sutures or grafts at maximum failure load. Additionally, the mode of failure was determined by visual inspection.

### Statistical analysis

A Mann-Whitney U-test was performed to evaluate group differences in maximum load, stiffness, and elongation at failure load. A Kruskal-Wallis test was used to test group differences in displacements at the three designated loading cycles. The significance level was set to *p* < 0.05. For all statistical analyses, SPSS 20.0 (IBM-SPSS, Armonk, NY, USA) was used. The Kolmogorov-Smirnov test was performed to determine if data were normally distributed.

## Results

### Cyclic loading test

A summary of displacements for the two techniques after 100, 500, and 1000 cyclic loading tests is presented in Table 1. Displacements of the TG at 100, 500, and 1000 cycles did not statistically differ from those of the TPS (*p =* 0.132, 0.589, and 0.485, respectively).

**Table 1:** Displacement during cyclic loading.

	Displacement (mm)		
	After 100 cycles	After 500 cycles	After 1000 cycles
TPS	1.5 ± 0.5	2.2 ± 0.6	2.7 ± 0.6
TG	1.0 ± 0.5	1.9 ± 0.8	2.4 ± 0.9

data are the mean ± standard deviation. TPS, transtibial pullout suture; TG, tendon graft.

### Load-to-failure testing

Load-to-failure testing was immediately performed after cyclic loading. The maximum load, stiffness, and elongation at failure load are given in Table 2. Compared to TPS, the TG technique had significantly lower elongation (*p* = 0.009) and higher stiffness (*p* = 0.008). The maximum load of TGs was significantly lower than that of the TPS (*p* = 0.015).

**Table 2:** Maximum load, stiffness, and elongation at failure load.

	Maximum load (N)	Stiffness (N/mm)	Elongation (mm)
TPS	258 ± 45^a^	14.9 ± 3.2^a^	24.3 ± 2.9^a^
TG	176.9 ± 46	26.6 ± 5.6	14.8 ± 4.0

data are the mean ± standard deviation. TPS, transtibial pullout suture; TG, tendon graft.

^a^ Significant differences between the two groups.

### Failure modes

**Fig 3:**
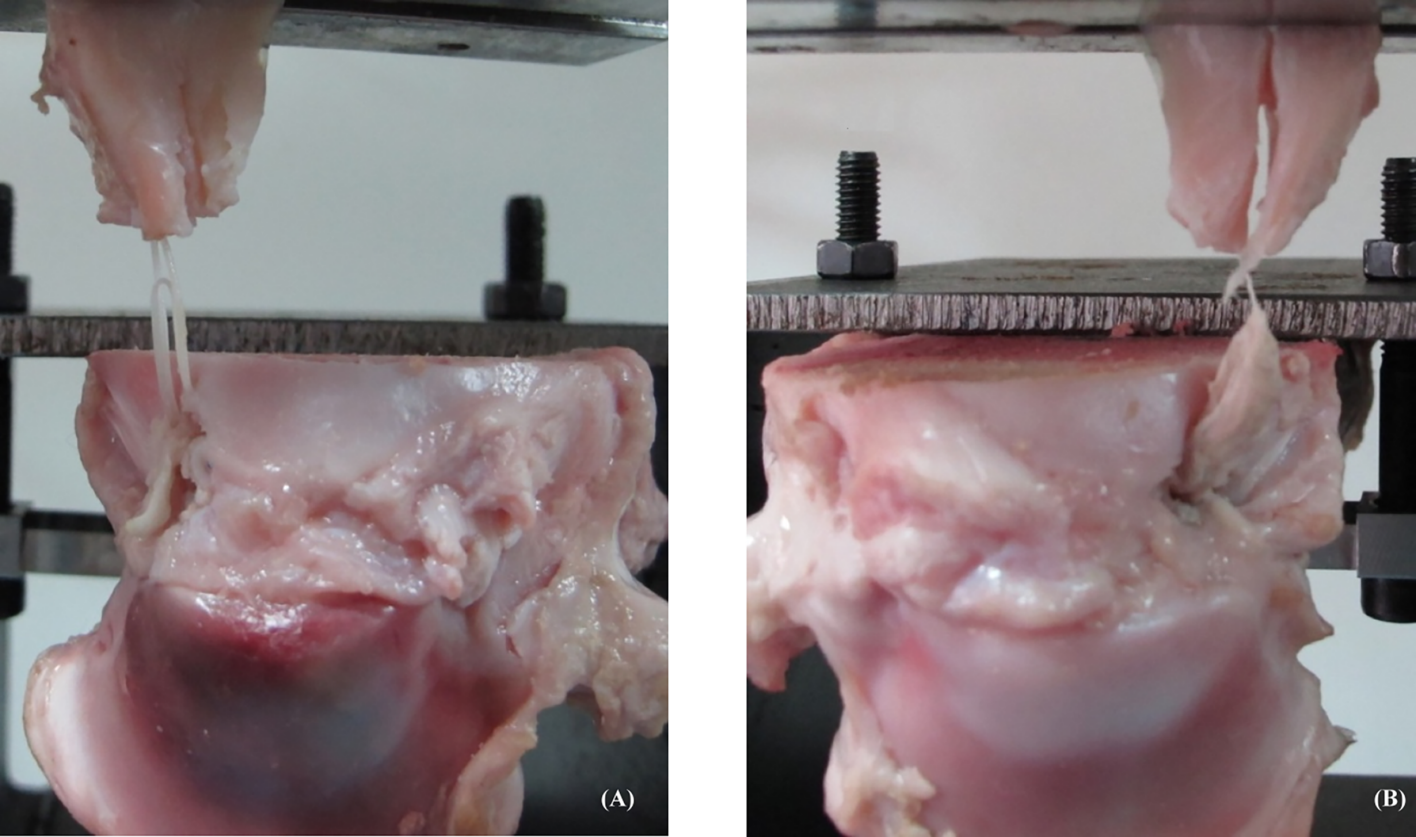
Failure modes of (A) the transtibial pullout suture (TPS) and (B) tendon graft (TG).

As to failure modes, all specimens of both TG and TPS failed due to sutures (Fig 3A) or grafts (Fig 3B) cutting through the meniscus along the sharp end of the incision site near the PMMR tear. No failure at the tibial fixation site was found.

## Discussion

In the present study, the TG group showed significantly less elongation, higher stiffness, and a lower maximum load in the load-to-failure tests compared to the TPS. The results supported our hypothesis that the TG technique could provide a stable construct and feasibility for clinical applications, the TG not only served as a spacer to maintain certain normal meniscus function but also bridged the gap to achieve anatomic repair. However, biological healing between the TG and meniscus or tibial plateau still requires further investigations in the future. In clinical situations, some PMMR tears are difficult to surgically repair if the radial tear is retracted far from the attachment site with a gap [39, 40]. For example, in most chronic tears, the remaining meniscal tissue is immobilized due to contracture or plastic deformation with poor tissue quality that makes anatomic repair infeasible [39]. Therefore, a meniscal transplant is usually recommended in addition to a partial meniscetomy. In particular, results of meniscus transplants are inconsistent, and there is concern over the relatively high rate of complications [45].

A successful pullout suture repair for PMMR tears greatly depends on stable fixation. Interestingly, Feucht et al. compared the biomechanical characteristics of four different types of suture techniques applied to repair PMMR tears. They illustrated that the modified Manson-Allen (MMA) suture technique had the least displacement (0.88 ± 0.11 mm) during cyclic loading and the highest failure load (335.2 ± 58.8 N) in the load-to-failure test in a porcine knee model [46]. LaPrade et al. demonstrated similar results using cadaveric knees, which revealed a mean displacement of 2.14 ± 0.65 mm after 1000 loading cycles and a maximum failure load of 325 ± 77 N. These biomechanical studies showed that the MMA technique had good biomechanical properties and provides strong tensile strength to resist mechanical failure. Furthermore, a previous study also showed that no. 2 FiberWire had preferential biomechanical characteristics for the MMR repair compared to the no. 2 PDS, no. 2 Ethibond, and 2-mm Fibertape [47]. Therefore, the MMA technique with no. 2 FiberWire was chosen as the control group.

Biomechanical characteristics of an ideal fixation construct include minimal elongation, high stiffness, and a high maximum load to maintain the repaired meniscus root in place during the biological healing process. Previous studies reported that time-zero displacements of transtibial pullout-repaired constructs in porcine knee models ranged 2.2~3.8 mm using different fixation methods after a cyclic loading test [7, 48]. In the present study, maximal displacements of the TPS and TG groups during the cyclic loading test were 2.7 ± 0.6 and 2.4 ± 0.9 mm, respectively, and no significant differences were found between the two groups. The “bungee effect” in this study was within the 3-mm cutoff value for displacement, which compromises the repair effect in a porcine knee model [49]. This indicates that both techniques can provide comparable stability under physiological loading conditions. In addition, since the maximal displacement of the TG group was smaller and further from the 3-mm cutoff value compared to the suture group, the graft group might be safer.

In the load-to-failure test, the TG group showed significantly lower elongation and higher stiffness but a lower maximum failure load compared to the TPS group. Among all of these biomechanical parameters, stiffness plays an important role in the ability to maintain the shape and stability of the repaired construct under mechanical loading conditions [47, 50].

All specimens in our study failed by the sutures or grafts cutting through the meniscus. No breakage of the suture or graft was noted in the study. There was also no failure at the tibial fixation site. These results possibly indicate that tibial fixation using a biointerference screw and augmented with a screw and washer is sufficiently strong for clinical applications. A recent biomechanical study showed that the weakest site of the repaired construct was at the suture-meniscus interface regardless of the material used [47]. Similarly, we found that the failure site of the TG group was at the graft-meniscus interface. In order to pass the 3-mm graft and enhance future healing, the peripheral incision should be created longitudinally at the red-red zone and be about 5 mm long. Recently, Ozeki et al. used an autogenous Achilles tendon graft augmented with BMP-7 [51] or synovial mesenchymal stem cells [52] to treat large meniscal defects in rats and shed light on regenerating the meniscus to prevent further degeneration of the knee cartilage. In the present study, the harvested flexor tendon was passed through the red-red zone of the medial meniscus and fixed into a bone tunnel to secure subsequent biological healing, such as anterior cruciate ligament reconstruction conducted by hamstring tendons [53]. The major limitation of this study would be its time-zero in vitro design. The main difference between this study and actual clinical situations was the use of a porcine knee model and a digital flexor tendon to simulate reconstruction of the human knee with a hamstring TG. Since human knees are generally difficult to obtain for practicing new techniques, an animal model was adopted to compare the initial mechanical characteristics of the two techniques in this study. Porcine knee models were often used in other studies to evaluate mechanical characteristics of the human knee due to their similarities in size, shape, and bone quality [54, 55] as well as the consistency of the tissue quality [47, 50, 56, 57]. Porcine flexor digitorum profundus tendons were used to simulate the human hamstring tendons, because the characteristics of these tissues are comparable [58, 59]. Although the study results of the TG group were promising, this might not reflect the *in vivo* forces to which the graft is subjected. These data indicated that under these conditions, it is necessary to obtain biomechanical and biological information *in vivo* with regard to the TG technique in the future.

## Conclusions

The superior biomechanical properties of the TG technique, such as decreased elongation and higher stiffness, could be beneficial for postoperative biological healing between the meniscus and tibial plateau. However, a slower rehabilitation program after the TG is recommended because of its relatively lower maximum failure load compared to the TPS technique. The TG technique is an entirely new concept for PMMR repair and could be an alternative for anatomic root repair in patients with a poor quality of the residual meniscus, a chronic tear with severe loss of tissue substance, or shortening of the meniscus root ligaments.

## Supporting information

S1 File The biomechanical test setup and statistical analysis methods. (PDF)
