# Supplementary material for: Novel technique for repairing posterior medial meniscus root tears using porcine knees and biomechanical study
Source: PLoS One. 2018 Feb 6;13(2):e0192027. doi: 10.1371/journal.pone.0192027 (PMC5800675; doi:10.1371/journal.pone.0192027)
Supplement: S1 File — (PDF) [file pone.0192027.s001.pdf]

Biomechanical study

All tests were performed at room temperature (25 ± 1 °C), and specimens were kept moist with saline solution. A material testing system (MTS Bionix 858, Eden Prairie, MN, USA) with a custom-made clamping device was used for tensile testing (Fig. 1). The clamping device was rigidly mounted onto the plate of the material testing machine, and the peripheral section of the medial meniscus was placed in a mechanical screw action clamp. In order to prevent meniscus slippage, the clamp was equipped with corrugated jaw faces (Fig. 2). To avoid interference with the stiffness analysis, the menisci were clamped 1 cm medial to the sutures or the TGs of the meniscus. After a preload of 2 N, all specimens were subjected to 1000 cycles of a load between 2 and 20 N at a rate of 0.5 Hz. Subsequently, specimens were loaded to failure at a rate of 0.5 mm/s. The number of cycles, displacement, and loads were recorded by MTS software. The following parameters were analyzed in all tests: (1) displacements after 100, 500, and 1000 cycles and (2) the maximum load, stiffness, and elongation at failure load. The displacement was defined as the differences in the crosshead position from the peak of the first cycle to the peak of cycle 100, 500, and 1000. The stiffness was calculated as the steepest slope of the load-deformation curve spanning 30% of the data points collected between load initiation and the maximum load at failure. Elongation was measured as the total displacement of sutures or grafts at maximum failure load. Additionally, the mode of failure was determined by visual inspection.

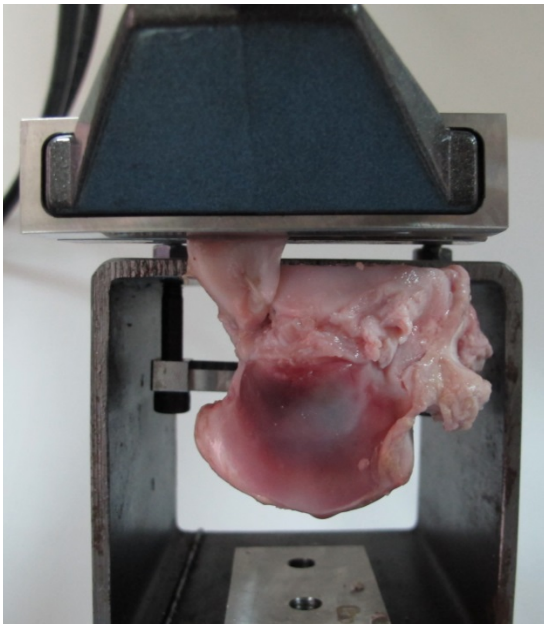

Fig. 1 Biomechanical test setup

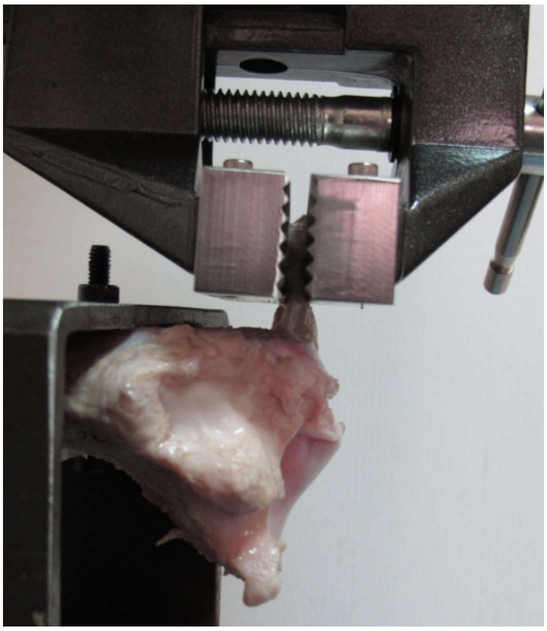

Fig. 2 The clamp was equipped with corrugated jaw faces

Statistical analysis

A Mann-Whitney U-test was performed to evaluate group differences in maximum load, stiffness, and elongation at failure load. A Kruskal-Wallis test was used to test group differences in displacements at the three designated loading cycles. The significance level was set to $p < 0.05$. For all statistical analyses, SPSS 20.0 (IBM-SPSS, Armonk, NY, USA) was used. The Kolmogorov-Smirnov test was performed to determine if data were normally distributed.

Independent T-test:

significant group differences at Max load, Stiffness, and Displacement at failure.

Group Statistics

	Group	N	Mean	Std. Deviation	Std. Error Mean
MaxLoad	Suture	6	258.4950	44.46535	18.15290
	Tendon	6	176.9300	46.41853	18.95029
Stiffness	Suture	6	14.9133	3.21835	1.31389
	Tendon	6	26.6217	5.62866	2.29789
Dis_failure	Suture	6	24.3217	2.88527	1.17791
	Tendon	6	14.8117	4.03361	1.64671

Independent Samples Test

		Levene's Test for Equality of Variances		t-test for Equality of Means						
									95% Confidence Interval of the Difference	
		F	Sig.	t	df	Sig. (2-tailed)	Mean Difference	Std. Error Difference	Lower	Upper
MaxLoad	Equal variances assumed	.523	.486	3.108	10	.011	81.56500	26.24198	23.09424	140.03576
	Equal variances not assumed			3.108	9.982	.011	81.56500	26.24198	23.07960	140.05040
Stiffness	Equal variances assumed	.640	.442	-4.423	10	.001	-11.70833	2.64700	-17.60621	-5.81045
	Equal variances not assumed			-4.423	7.954	.002	-11.70833	2.64700	-17.81852	-5.59814
Dis_failure	Equal variances assumed	.270	.614	4.697	10	.001	9.51000	2.02463	4.99884	14.02116
	Equal variances not assumed			4.697	9.055	.001	9.51000	2.02463	4.93421	14.08579

Cyclic

Mixed model ANOVA: no significant group differences was found

Between-Subjects Factors

		Value Label	N
Group	1	Suture	6
	2	Tendon	6

Descriptive Statistics

	Group	Mean	Std. Deviation	N
Dis_100cycle	Suture	1.4983	.48619	6
	Tendon	.9883	.52773	6
	Total	1.2433	.55224	12
Dis_500cycle	Suture	2.2367	.60305	6
	Tendon	1.9350	.77053	6
	Total	2.0858	.67823	12
Dis_1000cycle	Suture	2.6817	.60829	6
	Tendon	2.4200	.91686	6
	Total	2.5508	.75430	12

Estimates

Measure: MEASURE_1

Group	Mean	Std. Error	95% Confidence Interval	
			Lower Bound	Upper Bound
Suture	2.139	.263	1.553	2.725
Tendon	1.781	.263	1.195	2.367

Pairwise Comparisons

Measure: MEASURE_1

(I) Group	(J) Group	Mean Difference (I-J)	Std. Error	Sig.[a]	95% Confidence Interval for Difference[a]	
					Lower Bound	Upper Bound
Suture	Tendon	.358	.372	.359	-.471	1.186
Tendon	Suture	-.358	.372	.359	-1.186	.471

Based on estimated marginal means
